# Rare Diseases in Hospital Information Systems—An Interoperable Methodology for Distributed Data Quality Assessments

**DOI:** 10.1055/a-2006-1018

**Published:** 2023-05-16

**Authors:** Kais Tahar, Tamara Martin, Yongli Mou, Raphael Verbuecheln, Holm Graessner, Dagmar Krefting

**Affiliations:** 1 Department of Medical Informatics, University Medical Center Göttingen, Georg-August-University, Göttingen, Germany; 2 Centre for Rare Diseases, University Hospital Tübingen, Tübingen, Germany; 3 Chair of Computer Science 5, RWTH Aachen University, Aachen, Germany; 4 Medical Data Integration Center, University Hospital Tübingen, Tübingen, Germany

**Keywords:** Data quality, rare disease, distributed analysis, ontology, semantic interoperability, healthcare standards

## Abstract

**Background** Multisite research networks such as the project “Collaboration on Rare Diseases” connect various hospitals to obtain sufficient data for clinical research. However, data quality (DQ) remains a challenge for the secondary use of data recorded in different health information systems. High levels of DQ as well as appropriate quality assessment methods are needed to support the reuse of such distributed data.

**Objectives** The aim of this work is the development of an interoperable methodology for assessing the quality of data recorded in heterogeneous sources to improve the quality of rare disease (RD) documentation and support clinical research.

**Methods** We first developed a conceptual framework for DQ assessment. Using this theoretical guidance, we implemented a software framework that provides appropriate tools for calculating DQ metrics and for generating local as well as cross-institutional reports. We further applied our methodology on synthetic data distributed across multiple hospitals using Personal Health Train. Finally, we used precision and recall as metrics to validate our implementation.

**Results** Four DQ dimensions were defined and represented as disjunct ontological categories. Based on these top dimensions, 9 DQ concepts, 10 DQ indicators, and 25 DQ parameters were developed and applied to different data sets. Randomly introduced DQ issues were all identified and reported automatically. The generated reports show the resulting DQ indicators and detected DQ issues.

**Conclusion** We have shown that our approach yields promising results, which can be used for local and cross-institutional DQ assessments. The developed frameworks provide useful methods for interoperable and privacy-preserving assessments of DQ that meet the specified requirements. This study has demonstrated that our methodology is capable of detecting DQ issues such as ambiguity or implausibility of coded diagnoses. It can be used for DQ benchmarking to improve the quality of RD documentation and to support clinical research on distributed data.

## Introduction

As a result of digitization, the importance of data-driven research and innovation is continuously increasing, especially the electronic health record (EHR) is representing a rich source of information for clinical researchers. 1 The secondary use of patient data is considered to improve the quality of health care as well as clinical research. 2 3 4 Multisite research networks such as the project “Collaboration on Rare Diseases” (CORD-MI) 4 of the German Medical Informatics Initiative (MII) 2 connect various university hospitals to collect sufficient data for clinical research purposes. However, the integration of heterogeneous clinical data from different sources is challenging, 5 especially in the field of rare diseases (RDs) due to so-far lacking standardization of RD documentation in German hospitals. In Europe, the diseases that affect less than 5 in 10,000 people are defined as RDs. Typical RDs are complex chronic diseases and often life-threatening. 6 Furthermore, biomedical data aggregation from different sites raises concerns about the quality of integrated data due to the potential lack of semantic integrity and plausibility of the data provided in such research platforms. 7 As a consequence, this also raises concerns about the evidence level of findings and scientific outcomes derived from these data. 7 8 Data quality (DQ) remains therefore a main challenge for the secondary use of clinical data recorded in different health information systems. A sufficient high level of DQ as well as appropriate DQ assessment methods are needed to support the reuse of such heterogeneous data. 7 9 To solve these problems, various DQ frameworks have been proposed in the literature. 10 11 12 13 14 15 16 However, useful DQ assessments usually depend on the data set used, the domain of the planned use case, and the roles of the individual user of DQ reports. 9 10 Definition of specific indicators and DQ assessment methods is usually a complex but necessary task 17 because there is a need to consider domain and task dependencies as underlined in related works. 9 10 Despite the consensus on the definition and importance of DQ, standard DQ metrics and frameworks for specific use cases are still missing, especially in the field of RD documentation.

In CORD-MI, 20 German university hospitals and other partners from industry and research are committed to improving care and research in the field of RDs, as roughly 4 million people in Germany suffer from an RD of which in many cases little is known about the frequency, distribution, and course of these diseases. 4 Each university hospital in CORD-MI is a member of the MII. An essential aim of the MII is the implementation of data integration centers (DICs) at each university hospital. In CORD-MI, the DICs are responsible for the data extraction and integration from different heterogeneous sources within each hospital. The DICs, therefore, make the patient data on RDs available and reusable in an interoperable form that may be shared among other partner institutions. CORD-MI uses this infrastructure for improving care and research in the field of RD. For this purpose, CORD-MI use cases were designed to investigate various research questions regarding RDs across the research networks of MII. An accurate diagnostic representation of RD in the hospital information system (HIS) of participating hospitals is therefore required. One of the main objectives of CORD-MI is the implementation of the Orphacoding standard in existing workplace systems for offering physicians an easy way to encode RDs using Orphacodes (OCs). OCs are specific identification numbers assigned to each RD entity in the Orphanet nomenclature, 18 meeting the need of clinicians for an RD-specific coding system for diagnostic documentation and data sharing of RD patients. RD-specific coding with OCs is necessary to avoid any ambiguity in diagnosis documentation and to improve DQ as the majority of RDs are not represented in the International Classification of Diseases and Related Health Problems, 10th revision, German Modification (ICD-10-GM).

To increase the visibility of RD, the CORD-MI use cases aim at implementing observational studies on the treatment and documentation of selected RD diagnoses. These use cases include the monitoring of the care of RDs, also called “RD epidemiology and guidance for patients”, the analysis of the quality of care of patients with cystic fibrosis, the determination of the quality of care and long-term morbidity of treatable as well as treated RDs using the example of Phenylketonuria, and the quantification of psychiatric comorbidity for Fabry diseases. The ICD-10-GM is currently the only mandatory diagnosis coding system in German hospitals. This coding system is therefore the only system able to provide an epidemiological overview of at least the very few RD diagnoses explicitly and unambiguously represented in ICD-10-GM. Thus, a list of around 150 RD diagnoses were considered in the use case “RD epidemiology and guidance for patients”. The majority of these RD diagnoses were developed based on a study made by the University of Hamburg including only ICD-10-GM codes that can be assigned to a single RD or a group of RDs. 19 In MII, a national core data set (MII-CDS) 20 was developed as an information model to enable a harmonized exchange of health data. One of CORD-MI's key objectives is to provide solutions to improve clinical documentation and the quality of RD data recorded in heterogeneous HISs. In this paper, we therefore analyze the requirements and DQ issues surrounding RD documentation based on the MII-CDS. More specifically, the following research questions are answered: how can DQ be measured and assessed, which metrics are suitable for assessing the data of CORD-MI use cases, which methods and standards can be used to analyze the quality of data stored in distributed heterogeneous data sources, and what are the benefits of the developed methodology.

## Objectives

We propose an interoperable methodology for assessing the quality of RD data distributed over multiple organizations in the MII networks using DQ metrics that take the user and domain requirements into consideration. Our methodology comprises conceptual and software frameworks for DQ assessment. The conceptual framework – also called DQ concept – focuses on the theoretical foundation of used methods, concepts, and models, while the software framework provides technical guidance and a set of features to support the implementation and execution of the theoretical methods. In this context, we present our DQ concept that covers different dimensions of DQ as well as the resulting software framework that provides the features for calculating DQ metrics and generating local and cross-site reports on DQ. We further apply our implementation to synthetic data stored in different HISs. Finally, we show the generated results as a proof of concept and we share the current implementation of our methodology on GitHub. Our goal is to make the generated reports and developed tools available for other researchers in order to improve the quality of RD documentation.

## Methods

This section covers the design and implementation of an interoperable methodology that provides both conceptual and software frameworks for DQ assessment. We first define relevant terms, requirements, and DQ metrics used in our conceptual framework before we present the implemented software framework and validation methods in detail.

### Definition of Used Terms

Since the terms used in the literature on DQ often represent different abstraction levels of data management, we propose a detailed clarification of terms and definitions used in this paper as shown in Tables 1 and 2 .

**Table 1:** Definition of used terms

Term	Definition
DQ parameter	We use this term to denote valueless quantities of observation units, such as cases and patients. DQ Parameter do not allow any evaluation of DQ. However, appropriate DQ indicators are determined based on these parameters.
DQ indicator	DQ indicators are usually defined as dimensionless relative values or rates that are assigned to different categories of DQ, also called quality dimensions. In this paper, DQ indicators are expressed as percentage rates. A high value indicates high quality of data while a low value indicates possible DQ deficiencies.
Data item	This term is often used synonymously with the term data element or feature to specify a required atomic data property for a given use case. However, it is sometimes also used to describe a concrete value of this data property. In this paper, we use this term to denote an abstract specification of an atomic data property required for a given use case on the level of metadata (see Table 2 ).
Information model	We describe the smallest set of required data items for a specific use case as an information model (see Table 2 ). We would like to note that the term information model is also sometimes called data set, for example the MII core data set. 20
Data value	This term represents the concrete value of a data item within a given data set. It is also sometimes called a data field (see Table 2 ).
Data vector	We describe the available set of data values for a specific item as a data vector (see Table 2 ).
Data record	We use this term to describe a set of data values that is collected in one row to represent required information about an observation unit such as an individual patient or an individual case (see Table 2 ).
Subject record	A data set can also be divided into multiple records to capture information about involved subjects in a given study. We therefore introduce subject record as a set of data records required to capture information related to an individual subject such as inpatient or outpatient. In this context, we would like to note that the data of an individual patient as a study subject could be recorded in multiple data records, e.g., if a patient has various cases or diagnoses and the observation unit is case and not patient.
Data set	The set of multiple data vectors available for a given use case represents an instance of the used information model, which we denote as a data set (see Table 2 ). Since the extracted data are in a structural form, the concept in this context is also called data frame in programming environments such as R or Python.

Abbreviation: DQ, data quality.

**Table 2:** Usage of terms in this paper: The terms information model and data item refer to the specification of the data to be collected. They are therefore assigned to the metadata. The terms data value, data vector, and data record refer to the concrete data to be collected, while the term data set represents the entire instance of the used information model

### Data Quality Challenges and Requirements

The MII-CDS information model defines the semantics of required data items and, as consequence, provides the basis for developing harmonized DQ assessments across the MII including the CORD-MI network. The common data items specified in the MII-CDS are grouped into different modules such as Person, Treatment Case, and Diagnosis and are modeled as Fast Healthcare Interoperability Resources (FHIR) profiles related to the FHIR resources patient, encounter, and condition, respectively. The FHIR is a well-known standard describing data formats, resources, and the application programming interface for transferring EHR data between different software applications. This standard is developed by the Health Level 7 (HL7) international standard organization to achieve health care systems interoperability. 21 It is increasingly used for exchanging medical data for clinical research purposes. As a high outlier rate or missing rate in the required data items and values will raise concerns about the quality of scientific outcomes produced by CORD-MI use cases, the completeness and plausibility of data in the MII-CDS are therefore important aspects of DQ that are to be investigated in this study.

A feature of the large and diverse classes of RDs is their overall poor diagnostic representation in hospital documentation. Less than 10% of distinct RD diagnoses can be codified using a unique code in ICD-10-GM. Often, RDs are subsumed in unspecified ICD-10 codes that encode either both common and rare diseases or a single code for several distinct RDs, consequently rendering the majority of RDs invisible or indistinguishable. 22 23 University hospitals in CORD-MI, therefore, advanced the application of specific RD coding with OCs. The mandatory coding system ICD-10-GM and the (so far) voluntary OC are however inherently different in their organization and granularity. According to the possible relationships of the number of ICD-10-GM codes to the number of referring OCs, we can classify ICD-10-GM codes in four types represented as 1:1, n:1, 1:n, and 1:m. Codes of type 1:1 or n:1 are unambiguous, while ICD-10 codes of type 1:n or 1:m are ambiguous because they represent a group of RDs (1:n) or they are mixed into common diseases (1:m). Without additional OCs, it is impossible to determine the correct semantics of RD diagnoses coded using ICD-10-GM codes of type 1:n or 1:m. Such quality issues hamper the secondary use of EHR data for clinical research purposes for many RDs. Hence, the semantic unambiguity and completeness of RDs codification represent essential aspects that are to be covered in our DQ concept.

The Alpha-ID-SE, 24 published annually by the Federal Ministry of Health authority BfArM (Bundesinstitut für Arzneimittel und Medizinprodukte), provides a uniform and standardized mapping of the two coding systems, therefore allows coding of RD according to the ICD-10-GM, on the one hand, and the OC, on the other hand. While certainly not complete in covering all clinical entities and levels of the multihierarchical Orphanet nomenclature, Alpha-ID-SE provides selected OCs for more than 5,000 distinguished RDs. 25 In the past, only very few German hospitals have implemented Orphacoding due to lacking incentives for clinicians to dedicate valuable time for supplemental coding, in addition to shortcomings in the commercial coding software, and the lack of an exhaustive standardized mapping of relevant ICD-10-GM codes and OCs. 6 In those institutions that already introduced the coding system, Orphacoding has been characterized by tailored in-house solutions and workarounds. Therefore, while bearing the potential of a huge and necessary improvement on the visibility of RD patients, Orphacoding in the last years, if performed at all, has been highly heterogeneous in the disease scope of use, the quantity and plausibility of usage, and finally the quality of selected codes in relation to ICD-10-GM. The legislature has responded to the necessity of OCs for all RD patients and from 2023 onwards, 26 coding according to Alpha-ID-SE will become mandatory for all documentation of inpatient cases with RDs. Any services or treatments that require hospitalization are considered as inpatient cases. Alpha-ID-SE will therefore be the gold standard for required plausible mappings of ICD-10-GM and OCs in Germany. Evaluating if and to what extent the legal requirements of an appropriate and complete coding for all RD cases has been met will consequently become a challenge for local as well as regional and national DQ monitoring in hospitals. The basis for this quality control should therefore be a subset of ICD-10-GM codes within Alpha-ID-SE that exclusively code for RDs and consequently must be followed by an OC. In this work, we refer to these ICD-10-GM codes as tracer diagnoses.

Besides DQ metrics, interactive feedback loops to potential users are needed to improve the quality of collected RD data. Potential users in this context are for example medical documentation assistants, medical controlling staff, and data scientists. To establish an interactive DQ improvement process, specific DQ reports on detected DQ issues are required. The user should be able to select desired DQ indicators in order to define flexible DQ reports that focus on particular aspects of DQ. The generated reports should also provide adequate information to find the DQ violations and the causes of these violations. In this context, an interoperable and privacy-preserving solution for evaluating the DQ of RD documentation in distributed data sources is required.

### Definition of Data Quality Dimensions

**Fig. 1:**
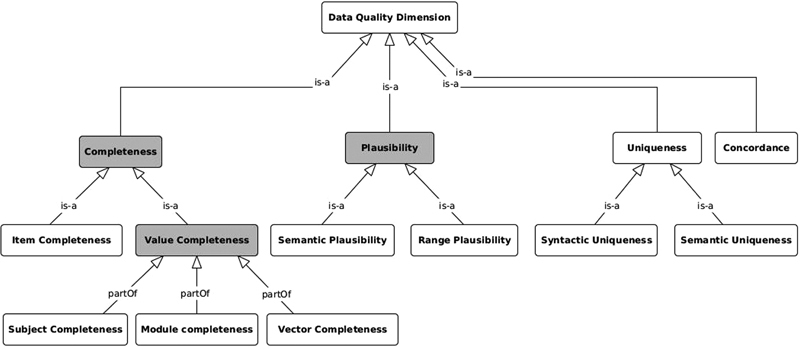
Ontology of used DQ concepts. The DQ dimensions defined by Kahn et al 11 are colored in gray. DQ, data quality.

Effective management of DQ in CORD-MI requires appropriate metrics and tools to assess the quality of data extracted from different HISs. In the literature, there is currently no consensus or standard framework for assessing the quality of clinical data. 10 11 12 13 14 Various DQ dimensions and related indicators have been proposed in previous related works. However, these metrics do not meet the specific requirements of CORD-MI use cases. In this work, two factors are considered for the selection of DQ metrics: (1) The selected dimensions should cover independent aspects of DQ, and (2) the definitions of indicators should reflect the individual requirements of implemented use cases. Based on the requirements specified above, the following dimensions have been selected for CORD-MI: completeness, plausibility, uniqueness, and concordance. Various synonyms and definitions are already provided in the literature for these dimensions. To avoid confusion, we propose the following definitions and subcategories in order to characterize the selected dimensions. Fig. 1 shows the ontological structure of used DQ concepts and their semantic relationships.

We used the harmonized DQ terminology developed by Kahn et al 11 to denote the core concepts of DQ, namely plausibility and completeness. This harmonized terminology is widely used in international frameworks such as DQA tool and OHDSI. 27 28 We further extended these core concepts to specifically address relevant aspects of DQ with the subcategories semantic plausibility, range plausibility, and item completeness as the ontology in Fig. 1 shows. Moreover, our DQ concept differentiates uniqueness from plausibility to avoid confusion. We focus on semantic uniqueness, which is necessary for the secondary use of clinical data as described in Section “Data Quality Challenges and Requirements”. In contrast, Kahn et al 11 define uniqueness as a subcategory of plausibility called “uniqueness plausibility,” which seeks to determine the frequency of duplicated objects in a given data set. The conformance metrics proposed by Khan et al were not applied in our DQ concept because the FHIR standard implemented in this work already supports conformance checks; instead, we used an additional dimension called concordance, which is important for cross-site DQ assessments and is explained below.

#### Completeness

Completeness represents the degree to which the relevant data are available. This dimension should be therefore evaluated by means of missing checks. There is wide consensus on this concept in the literature. 10 11 12 However, besides the well-known value completeness that measures the completeness of data sets for given information models, we introduce here the item completeness (see Fig. 1 ), which is a quality issue that specifically arises from multisite and multisystem data collections: the completeness of the local information models regarding an external reference model such as — in our case — the FHIR profiles of the MII-CDS. While item completeness investigates DQ issues on the level of metadata, value completeness focuses on the data itself. We distinguish these two main subcategories of the completeness dimension because resulting DQ issues would require different actions on different targets: value completeness must be accomplished by those who generate the data, while item completeness must be accomplished by configuring the EHR data entry mask or the Extract-Transform-Load (ETL) and FHIR mapping processes. Clinical data sets usually comprise multiple data vectors and data modules. Data vectors record individual data items, while data modules collect data item groups such as the FHIR resources patient or encounter. We therefore introduce two different categorial parts of value completeness as shown in Fig. 1 : (1) vector completeness that focuses on the completeness of individual data vectors such as OC, and (2) module completeness which evaluates the completeness of specific data modules such as the case module described in Section “Completeness Indicators”. Similar to data modules, a given data set can also be divided into multiple subject records as described in Table 1 . We therefore introduce a third categorial part of value completeness called subject completeness that investigates the completeness of specific subject records in a given data set such as inpatient or outpatient records.

#### Plausibility

Various synonyms are provided in the literature to describe this dimension such as correctness 10 and consistency. 12 The concept always describes deviations from expected values. However, there are three reasons why we think the term “plausibility” best describes the concept. First, consistency checks in logic and set theory are usually carried out by means of inference models. 29 These consistency checks require therefore a formal representation using results from computable set theory, which is time-consuming and typically not available for RD data. 30 Second, the assessment of consistency in mathematics requires a mathematical proof also called consistency proof. 31 Third, we can analyze the plausibility of acquired data using computer-based assessments. However, the correctness of these data can only be judged by domain experts. We have therefore avoided using the terms “consistency” and “correctness” to characterize this concept. In particular, we use this quality dimension to evaluate the plausibility of recorded data regarding the absence of outliers and semantic contradictions. The semantic and temporal dependencies of data values should be therefore evaluated using plausibility checks. In this paper, we differentiate between two subcategories of plausibility: (1) semantic plausibility and (2) range plausibility (see Fig. 1 ). Semantic plausibility represents DQ issues resulting due to violation of semantic models such as reference lists and ontologies, while range plausibility reflects the contravention of expected limits, for example, the violation of expected statistical distributions in a data vector.

#### Uniqueness

Uniqueness represents the degree to which the data are free from ambiguity and duplications. This dimension is very important for the reuse of collected data for new research purposes. We differentiate between two independent facets of uniqueness: (1) syntactic uniqueness, which investigates duplicated patient data with duplicated identities as well as duplicated events such as case or lab values and (2) semantic uniqueness, which focuses on the unambiguousness of the semantic interpretation. Ontologies and classification systems are usually used to semantically annotate clinical data. The accuracy of such annotations affects the quality of the data and their semantic interpretation. A detailed specification is therefore necessary to avoid the ambiguity of coded RD diagnoses. Moreover, the use of OCs representing specific RDs will improve the quality of RD documentation and captured data, especially on the level of semantic uniqueness. As described in Section “Data Quality Challenges and Requirements”, diagnostic information on RDs is often ambiguous. The ICD-10-GM code Q28.21, for example, is of type 1:n and represents different RDs that include cerebral arteriovenous shunt and cranial dural arteriovenous fistula as shown in Table 3 . Consequently, it is impossible to determine the right diagnosis using such ICD-10-GM codes, although we can state that it is a patient with RD. Another example of an ambiguous code is the ICD-10-GM code E03.0, which is of type 1:m and therefore represents common diseases as well as RDs (see Table 3 ). This lack of semantic unambiguousness makes the reuse of RD data very difficult. On the contrary, each of these RDs has a unique OC. Hence, it is necessary to use OCs in order to identify patients with RDs in EHR data.

**Table 3:** Exemplary RD diagnoses from Alpha-ID-SE terminology version 2022 (first four columns) extended with two columns to classify the type of relationship between ICD-10-GM codes and OCs as well as the type of diagnoses

Alpha-ID	ICD-Primary Code	Orphcode	Label	Type of Relationship	Type of Diagnosis
I95787	E84.80	586	Cystic fibrosis	n:1	UTD
I18534	E84.9	586	Cystic fibrosis	n:1	UTD
I125102	K62.7	70475	Radiation proctitis	1:1	UTD
I98990	Q28.21	46724	Cerebral arteriovenous shunt	1:n	ATD
I119801	Q28.21	97339	Cranial dural arteriovenous fistula	1:n	ATD
I127608	E03.0	95716	Familial thyroid dyshormonogenesis	1:m	AD
I2008	E03.0		Congenital goiter	1:m	AD
I95978	E03.0		Congenital diffuse goiter	1:m	AD
I75872	E03.0		Congenital non-toxic goiter	1:m	AD

Abbreviations: AD, ambiguous Diagnosis; ATD, ambiguous Tracer Diagnosis; ICD-10-GM, International Classification of Diseases and Related Health Problems, 10th revision, German Modification; OC, Orphacodes; RD, rare disease; UTD, unambiguous Tracer Diagnosis.

#### Concordance

There are various definitions of concordance reported in the literature. 32 33 34 According to Snowden et al, 35 the conceptualization and use of the term concordance differ between the various disciplines because the expression of this concept depends on the political, professional and legal drivers of these disciplines. However, the agreement aspect is a common understanding between different domains. In the context of databases, this concept usually describes the comparison of the data values of a given data set to a local reference source in order to assess the reliability of analyzed data values, for example, to investigate if there is concordance between the data values stored in EHR and another local source. In this paper, we focus however on the concordance of relevant DQ parameters instead of data values. External references are, therefore, required for investigating the level of agreement with the literature and national references on an aggregated level. The results of such a concordance analysis are also used as presented by Iyen-Omofoman et al 36 to evaluate representativeness in comparison with national databases. We take use of measurements provided by the literature and explore the extent to which resulting DQ metrics in one DIC are concordant with external results found in the literature and national references. Hence, new DQ indicators are required to compare local DQ results to those of external data sources and to determine whether they are contradictory (see Section “Concordance Indicator”).

### Definition of Data Quality Indicators

CORD-MI use cases require DQ indicators to assess the quality of data quantitatively. Suitable DQ metrics are therefore derived from the dimensions introduced above. In this section, we give definitions of used DQ indicators (I1,..,I10) and related parameters (P1,…,P25) that are listed in Tables 4 and 5 , respectively. We refer to Table 4 for the mathematical definition of the parameters and give only the equations for the indicators within the paragraphs for better readability. Regarding the tracer diagnoses specifically relevant for CORD-MI, there is no information in the Alpha-ID-SE terminology about whether a given ICD-10-GM code is a tracer diagnosis or not and whether this code specifies an ambiguous RD diagnosis as explained above. We therefore extended this system with required classifications as shown in Table 3 to make it useful for assessing the completeness of Orphacoding and unambiguity of RD cases. A formal list of tracer diagnoses 37 38 was automatically extracted from the Alpha-ID-SE 24 terminology as described under “Implementation of the Software Framework and Data Quality Assessment Methods”. This list provides a classification of tracer diagnosis into unambiguous tracer diagnoses of type (n:1 or 1:1) and ambiguous tracer diagnoses of type (1:n).

**Table 4:** DQ parameters displayed in the generated reports

No.	Name	Abr.	Definition	Mathematical equation
P1	Mandatory data items	im	Number of data items that are mandatory in a given information model defined as a set of data items	Given an information model M, ai = 1 if i-th data item in M is mandatory else 0. P1: *im* *=* *Σ ai*
P2	Missing mandatory data items	im_misg	Number of mandatory data items that are absent in a given data set	Given a data set S following the information model M, bi = 1 if i-th mandatory data item in M is absent in S, else 0. *P2: im_misg* *=* *Σ bi*
P3	Mandatory data values	vm	Number of possible mandatory data values in a given data set	Given a data set *S* with *n* mandatory data items and m data records. *P3: vm* *=* n * m
P4	Missing mandatory data values	vm_misg	Number of mandatory data values that are empty or NA in a given data set	Given a data set S, cij = 1 if i-th data value of mandatory data item j in *S* is empty or NA else 0. *P4: vm_misg* *=* *Σ Σ cij*
P5	Inpatient cases	ipatCase	Number of inpatient cases in a given hospital	Given a data set S of all cases in a hospital including the data item “encounter class” that captures the type of recorded cases, di = 1 if the i-th unique case in S is of type inpatient else 0. *P5: ipatCase* *=* *Σ di*
P6	Inpatients	ipat	Number of inpatients in a given hospital. This number is equal to the number of subject records (s) because in our study we consider inpatients as subjects	Given a data set S as introduced in P5 that also includes the data item “patient ID”, ei = 1 if the i-th unique patient in S has a related case of type inpatient else 0. *P6: ipat* *=* *Σ ei*
P7	Incomplete inpatient records	ipat_inc	Number of incomplete inpatient records (cf. P6). This number is equal to the number of incomplete subject records (s_inc)	Given a data set S as introduced in P6, fi = 1 if the i-th inpatient record in S has at least one missing data value of a mandatory data item else 0. *P7: ipat_inc* *=* *Σ fi*
P8	Mandatory data values in the case module	vm_case	Number of data values required for recording all mandatory items of the case module, i.e. the treatment case and diagnosis profiles of the MII-CDS	Given a data set S with p data records following an information model M that requires q mandatory data items for the definition of the case module C. *P8: vm_case* *=* p * q
P9	Missing data values in the case module	vm_case_misg	Number of data values that are absent for recording the mandatory data items of the case module (cf. P8)	Given a data set S with a case module C as defined in P8, hij = 1 if ith data value of data item j in C is empty or NA else 0. *P9: vm_case_misg* *=* *Σ Σ hij*
P10	Data values selected for outlier detection	v_slc	Number of data values checked for outliers in a given data set	Given a data set S including a subset S' of selected data vectors with n' items and m' records. P10: v_slc= n' * m'
P11	Outliers	v_ip	Number of detected outliers (implausible data values) in a given data set	Given a data set S including a subset S' of selected data vectors, lij = 1 if i-th data value of data item j in S' is an outlier else 0. *P11: v_ip* *=* *Σ Σ lij*
P12	Tracer diagnoses	icd_tracer	Number of diagnoses with ICD-10 codes that exclusively code RD in a given data set	Given a list of tracer diagnoses L and a data set S including a data vector of ICD-10 codes v. For all data values x in v, yi =1 if the i-th data value xi ∈ L else 0. *P12: icd_tracer* *=* *Σ yi*
P13	Missing Orphacodes	oc_misg	Number of diagnoses with ICD-10 code indicating an RD, where no Orphacode is present	Given a data set S including among other data vectors, the vectors vi and vj, vi captures ICD-10 codes while vj records OCs. pk = 1 if k-th data value in vi is a tracer diagnosis and the kth data value in vj is missed else 0. *P13: oc_misg= Σ pk*
P14	Checked links	link	Number of ICD-10-GM/OC links in a given data set	Given a data set S including the data vectors vi and vj, vi captures ICD-10 codes while vj records OCs. qk = 1 if k-th data value in vi and the k-th data value in vj are not missed else 0. *P14: link= Σ qk*
P15	Implausible links	link_ip	Number of ICD-10-GM/OC links not present in the respective Alpha-ID terminology in a given data set	Given a data set S as defined in P14, rk = 1 if the combination of k-th data value in vi and k-th data value in vj is implausible else 0. *P15: link_ip* *=* *Σ rk*
P16	RD cases	rdCase	Number of cases that are coded using OCs or ICD-10-GM/OC links or ICD-10 codes from the list of tracer diagnoses	Given a data set S with n1 cases coded using individual OCs, n2 cases coded using ICD-10-GM/OC links and n3 cases coded using individual ICD-10 tracer diagnoses (see P12). *P16: rdCase* *=* *n1* *+* *n2* *+* *n3*
P17	Ambiguous RD cases	rdCase_amb	Number of RD cases coded using ambiguous ICD-10-GM/OC links or tracer diagnoses	Given a data set S with RD cases as defined in P16, si = 1 if the i-th RD case in S is ambiguous else 0. *P17: rdCase_amb* *=* *Σ si*
P18	Duplicated RD cases	rdCase_dup	Number of duplicated RD cases in a given data set	Given a data set S including RD cases as defined in P16, ti = 1 if the i-th RD case is duplicated else 0. *P18: rdCase_dup* *=* *Σ ti*
P19	Tracer cases	tracerCase	Number of RD cases coded at least using an ICD-10 code from the list of tracer diagnoses	Given a list L and a data set S as defined in P12, ui =1 if the i-th case in S is at least coded using an ICD-10 code ui and ui ∈ L else 0. *P19: tracerCase* *=* *Σ ui*
P20	Orpha cases	orphaCase	Number of RD cases coded at least using an OC	Given a data set S, zi =1 if the i-th case in S is at least coded using an OC else 0. *P20: orphaCase* *=* *Σ zi*
P21	RD cases relative frequency	rdCase_rel	Relative frequency of RD cases normalized to 100,000 inpatient cases	Given a data set S with the DQ parameters ipat as defined in P6 and rdCase as defined in P16. *P21: rdCase_rel* *=* (rdCase*100,000)/ipat
P22	Tracer cases relative frequency	tracerCase_rel	Relative frequency of tracer cases normalized to 100,000 inpatient cases	Given a data set S with the DQ parameters ipat as defined in P6 and tracerCase as defined in P19. *P22: tracerCase_rel* *=* (tracerCase*100,000)/ipat
P23	Orpha cases relative frequency	orphaCase_rel	Relative frequency of Orpha cases normalized to 100,000 inpatient cases	Given a data set S with the DQ parameters ipat as defined in P6 and orphaCase as defined in P20. *P23: orphaCase_rel* *=* (orphaCase*100,000)/ipat
P24	Minimal tracer cases in reference values	tracerCase_rel_min	Minimal relative frequency of tracer cases normalized to 100,000 inpatient cases found in the literature	Given a set T of relative tracer cases reported in the literature. *P24: tracerCase_rel_min* *=* *min(T)*
P25	Maximal tracer cases in reference values	tracerCase_rel_max	Maximal relative frequency of tracer cases normalized to 100,000 inpatient cases found in the literature	Given a set T of relative tracer cases reported in the literature. *P25: tracerCase_rel_max* *=* *max(T)*

Abbreviations: DQ, data quality; ICD-10-GM, International Classification of Diseases and Related Health Problems, 10th revision, German Modification; OC, Orphacodes; RD, rare disease.

**Table 5:** Data quality indicators (DQIs) displayed in the generated reports

No.	DQI	Abr.	DQ Category	Mathematical equation
I1	Item Completeness Rate	dqi_co_icr	Item Completeness	*I1: dqi_co_icr* *=* *(im-im_misg)/im*
I2	Value Completeness Rate	dqi_co_vcr	Value Completeness	*I2: dqi_co_vcr* *=* *(vm-vm_misg)/vm*
I3	Subject Completeness Rate	dqi_co_scr	Subject Completeness	*I3: dqi_co_scr* *=* *(s-s_inc)/s*
I4	Case Completeness Rate	dqi_co_ccr	Module Completeness	*I4: dqi_co_ccr* *=* *(vm_case-vm_case_misg)/vm_case*
I5	Orphacoding Completeness Rate	dqi_co_ocr	Vector Completeness	*I5: dqi_co_ocr* *=* *(icd_tracer-oc_misg)/icd_tracer*
I6	Orphacoding Plausibility Rate	dqi_pl_opr	Semantic Plausibility	*I6: dqi_pl_opr* *=* *(link-link_ip)/link*
I7	Range Plausibility Rate	dqi_pl_rpr	Range Plausibility	*I7: dqi_pl_rpr* *=* *(v_slc-v_ip)/v_slc*
I8	RD Case unambiguity Rate	dqi_un_cur	Semantic Uniqueness	*I8: dqi_un_cur* *=* *(rdCase-rdCase_amb)/rdCase*
I9	RD Case Dissimilarity Rate	dqi_un_cdr	Syntactic Uniqueness	*I9: dqi_un_cdr* *=* *(rdCase-rdCase_dup)/rdCase*
I10	Concordance with Reference Values from Literature	dqi_cc_rvl	Concordance	*I10: dqi_cc_rvl* *=* *1 if tracerCase ∈ [traceCase_rel_min, tracerCase_rel_max] else 0*

Abbreviation: RD, rare disease **.**

#### Completeness Indicators

##### Item Completeness Rate (dqi_co_icr)

This indicator assesses the metadata completeness of a given data set and evaluates whether mandatory data items (im), for example “ICD_Code”, of the information model were collected. The mandatory data items are specified using the FHIR profiles 39 of the MII-CDS. The absence of a mandatory data item in the metadata of a given data set is considered as a missing mandatory data item (im_misg): *dqi_co_icr* *=* *(im-im_misg)/im (I1)* .

##### Value Completeness Rate (dqi_co_vcr)

While dqi_co_icr evaluates the completeness of the mandatory data items, the value completeness rate focuses on the completeness of recorded data itself. This indicator therefore shows whether all data values, for example “E75.2”, of existing mandatory data items such as “ICD_Code” are collected. We describe such data as mandatory data values (vm). The absence of an individual value detected in a given data vector of a mandatory data item is considered as a missing mandatory data value (vm_misg). Missing data values due to missing data items are not considered. Hence, this indicator represents the proportion of uncertainty due to missing data values detected by existing data vectors of mandatory items: *dqi_co_vcr* *=* *(vm-vm_misg)/vm (I2)* .

We would like to emphasize that this indicator cannot detect missing FHIR resources, such as a second diagnosis that would be captured as a second FHIR resource (condition), as these are optional in the information model. Furthermore, coded missing values are not considered in this indicator, as they could be arbitrary nonplausible values due to the heterogeneous primary systems and code systems allowed in FHIR. Such coded missing values are to be detected in the range plausibility rate (dqi_pl_rpr), as these are defined for the individual items.

##### Subject Completeness Rate (dqi_co_scr)

We introduce this indicator to evaluate the completeness of subject records as defined in Table 1 . This indicator therefore shows whether all mandatory data values in existing subject records (s) are collected. Subject records with at least one missing data value detected by an existing data vector of a mandatory data item are considered as incomplete subject records (s_inc): *dqi_co_scr* *=* *(s-s_inc)/s (I3)* .

We would like to note that duplicated subject records and missing data values due to missing data items are not considered in this indicator. In our case, we consider inpatients as subjects. The number of subject records is therefore equal to the number of inpatients (ipat), and as consequence, the number of incomplete subjects is also equal to the number of incomplete inpatient records (ipat_inc) as shown in Table 4 .

##### Case Completeness Rate (dqi_co_ccr)

This indicator assesses the completeness of data values required for recording the case module in a given data set. Case refers here to mandatory data items for a case in CORD-MI and encompasses both MII-CDS modules treatment case and diagnosis. Case completeness is therefore an instance of module completeness. The case module includes a set of data vectors related to the following data items: patient ID, encounter ID, encounter status, encounter class, admission date, discharge date, diagnosis code, diagnosis role, and diagnosis date. dqi_co_ccr evaluates whether all required data values for mandatory data items (vm_case) are present. In contrast to *dqi_co_vcr, dqi_co_ccr* also considers missing values of mandatory data items (vm_case_misg) even if not available in the local information model: *dqi_co_ccr* *=* *(vm_case-vm_case_misg)/vm_case (I4)* .

##### Completeness Rate of Orphacoding (dqi_co_ocr)

This indicator evaluates whether all cases with tracer diagnoses (icd_tracer) are coded using OCs. We used the formal list of tracer diagnoses as a reference for detecting available tracer diagnoses and missing OCs (oc_misg) in a given data set: *dqi_co_ocr* *=* *(icd_tracer-oc_misg)/icd_tracer (I5)* .

We would like to emphasize that we cannot detect missing OCs in ICD-10-GM codes of type 1:m, as it would require further clinical evaluation to determine if the code was used to code an RD or a common disease.

#### Plausibility Indicators

##### Plausibility Rate of Orphacoding (dqi_pl_opr)

This indicator assesses the semantic plausibility of links between ICD-10-GM and OC, that is, concurrent codes in the Diagnosis module. All semantic links available in a given data set (link) are evaluated using the standard Alpha-ID-SE terminology. An implausible ICD-10-GM/OC link (link_ip) is defined as a combination of ICD-10-GM and OC that is absent in the Alpha-ID-SE terminology valid at the time of coding. The valid version of the used terminology depends on the data set itself. For example, the Alpha-ID-SE version published in 2022 should be used for analyzing data collected in 2022: *dqi_pl_opr* *=* *(link-link_ip)/link (I6)* .

##### Range Plausibility Rate (dqi_pl_rpr)

This indicator evaluates the plausibility of data values in selected data vectors (v_slc). In this context, outliers, that is, implausible values (v_ip), are defined as data values within the selected values (v_slc) that do not meet the user expectations (such as an age value over 115):

*dpi_pl_rpr = (v_slc-v_ip)/v_slc (I7).*

#### Uniqueness Indicators

##### Unambiguity Rate of RD Cases (dqi_un_cur)

This indicator assesses the semantic uniqueness of coded RD cases (rdCase) in a given data set. All cases with documented ICD-10-GM/OC links or individual OCs or individual ICD-10-GM codes from the list of tracer diagnoses are considered as RD cases. The unambiguousness of RD cases is evaluated using an appropriate algorithm, which uses the Alpha-ID-SE terminology and the list of tracer diagnoses as references for the classifications of RD diagnoses. Ambiguous RD cases (rdCase_amb) are cases coded using ambiguous ICD-10-GM/OC links or tracer diagnoses of type 1:n:

*dqi_un_cur* *=* *(rdCase-rdCase_amb)/rdCase (I8).*

We would like to note that cases with documented common diseases in the primary diagnosis and RD in the secondary diagnosis are also considered as RD cases. The primary diagnosis is the one responsible for causing the patient's hospitalization, while secondary diagnoses are complications that already coexist with a primary diagnosis or are developed during the inpatient hospitalization.

##### Dissimilarity Rate of RD Cases (dqi_un_cdr)

This indicator evaluates the syntactic uniqueness of recorded RD cases. A high proportion of duplicate cases (rdCase_dup) in the data set may be due to systematic double documentation or to a systematic error in the used information system:

*dqi_un_cdr = (rdCase-rdCase_dup)/rdCase (I9).*

#### Concordance Indicator

##### Concordance with Reference Values from Literature (dqi_cc_rvl)

RD cases that are coded at least with a tracer diagnosis are called tracer cases. This indicator is a measure, if the relative frequency of reported cases including a tracer diagnosis — here called tracer cases (tracerCase) — lies in the range found in the literature. The relative frequency of tracer cases (tracerCase_rel) is the ratio of coded tracer cases to the total inpatient cases this year in a given hospital normalized to 100,000 inpatient cases. The indicator *dqi_cc_rvl* evaluates therefore if there is concordance between the relative frequency of tracer cases measured locally in a given DIC and the relative frequency of tracer cases provided by literature references. If there is concordance with the literature the indicator output will be 1, else 0. We define as concordance limits the minimal (traceCase_rel_min) and maximal (traceCase_rel_max) values found in the literature:

*dqi_cc_rvl* *=* *1 if tracerCase ∈ [traceCase_rel_min, tracerCase_rel_max] else 0 (I10).*

We acknowledge that the choice of the limits is disputable and that statistical measures such as the standard deviation or the quartiles might have been expected here. But in the current situation where only one reference is available, it is our opinion that concordance should be defined generously until more reference data are available that allows quantitative statistics.

Lehne et al 40 investigated 143 tracer diagnoses required for CORD-MI and found that the relative frequency of tracer cases measured using the German National Case Statistics (NCS) was tracerCase_rel_mean = 294.8 per 100,000 cases, while this frequency rate is 3.14 times higher in university hospitals. We cannot therefore use tracerCase_rel_mean as a reference for our concordance analysis; instead, we take the provided consistent pattern of ratios between the relative frequency of tracer cases across different ICD-10-GM chapters to NCS ranging from a minimum (min) of 2.01 for diseases of the nervous system to a maximum (max) of 6.28 for the skin subcutaneous tissue as aggregated reference levels. We use these ratios to define a tolerance interval *I* *=* [min*294.8, max*294.8] for assessing the concordance of tracer cases at each hospital, resulting in frequencies of tracer cases that lie in the interval *I* *=* *[593, 1851]* fulfilling our concordance criterion.

### Implementation of the Software Framework and Data Quality Assessment Methods

As proof of concept, we provide an open-source implementation of our software framework that can be executed locally or in distributed environments. Our tools include (1) An R package for DQ assessment and reporting, (2) R scripts as an exemplary implementation specific for CORD-MI, (3) a tracer diagnoses list, (4) Personal Health Train (PHT) for distributed DQ assessments, (5) FHIR tools, and (6) a Docker file for local execution. All developed tools and generated DQ reports are available on GitHub. 37 41 42 In the following, we introduce these implemented tools.

Our DQ concept introduced above is implemented as an R package that provides reusable methods for calculating DQ metrics and generating user-defined DQ reports. The developed package is used as a software framework called DQ library 41 to develop reporting scripts for DQ assessment. 38 Essentially, this software library has a modular design that allows the user to select desired parameters and indicators as well as to generate specific reports that include selected metrics and detected DQ issues. Using this framework, we developed tools for locally and cross-institutional analysis of DQ in CORD-MI. 37

To make the Alpha-SE-ID terminology useful for assessing the OC indicators presented above, we extended this terminology with required classifications as shown in Table 3 . A formal list of tracer diagnoses was therefore automatically generated using a computer-based classification approach that identifies tracer diagnoses listed in the Alpha-ID-SE terminology and classifies them into unambiguous and ambiguous tracer diagnoses. We used this list as a reference for detecting tracer diagnoses available in the analyzed data and evaluating the quality of RD documentation. The generated reference list can be downloaded from the GitHub repository. 37

**Fig. 2:**
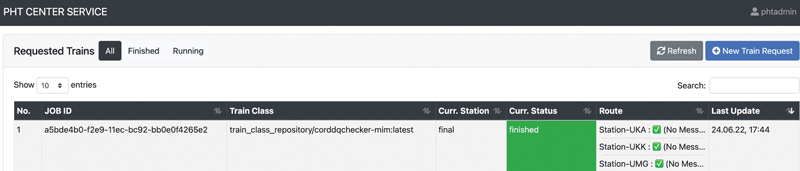
Train route for distributed DQ assessments over the three German hospitals (UKA, UKK, and UMG). DQ, data quality; UKA, University Hospital RWTH Aachen; UKK, University Hospital Cologne; UMG, University Medical Center Göttingen.

To enable cross-site reporting on DQ, a distributed DQ analysis was implemented using PHT. 43 44 PHT is an infrastructure to support distributed data analytics of medical health data, while the data remain under the control of the data holders. There are two main concepts in PHT, i.e., station and train. Stations are the data holding nodes that expose data in a discoverable format, define data source interfaces to execute queries, and execute analytical tasks in a secure environment. Trains are the encapsulated analytical tasks (including algorithms, queries, and intermediate results) based on containerization technologies and travel from one station to the next to update the results. Between stations, the results inside containers are encrypted to prevent manipulation or disclosure. PHT provides a core component in its architecture for researchers, so-called Central Services, that allows researchers to define and send train job requests, to monitor the execution process (as shown in Fig. 2 ), and to view the results. For distributed DQ analysis, we implemented the algorithms as a Docker image and ran it on the PHT platform in a distributed way. The developed PHT image is available on GitHub repository. 37

To enable interoperable DQ assessments, we developed a FHIR interface using the fhircrackr package 45 and applied it to different FHIR data sets distributed across multiple hospitals. The used data sets follow the MII-CDS and contain randomly introduced DQ issues as described under “Experiment Settings for Distributed Data Quality Assessments”. The so-called HAPI FHIR server 46 was installed for storing these synthetic FHIR data sets at each hospital. 42 The developed interface ensures an interoperable execution of our DQ methodology that does not depend on local configuration or HIS architectures. In Section “Experiment Settings for Distributed Data Quality Assessments”, we present the standardized data provision using FHIR and the used distribution for evaluating our methodology across multiple hospitals.

Finally, we would like to emphasize that our solution also provides an Excel and CSV interface for importing tabular data as well as a Docker file for local execution (see GitHub repository 37 ). This enables an easy execution of local DQ assessments, in order, for example, to evaluate the quality of data directly extracted from HIS data sources using exports in Excel or CSV formats (before the transformation to FHIR format). In Section “Experiment Settings for Local Data Quality Assessment”, we present the experimental validation for local DQ assessments.

### Experiment Design and Validation Methods

We used precision and recall as metrics to validate the developed methodology and software solutions for DQ assessment. Precision describes the rate of detected DQ issues that were correct, while recall represents how many of existing DQ issues were detected. We measured precision and recall by comparing the obtained results with the distribution of DQ issues shown in Table 6 . In the following, we present the used experimental settings and data sets for validating the implemented DQ assessments.

**Table 6:** Distribution of DQ issues in synthetic data over the three German hospitals (UKA, UKK, and UMG)

DQ Issues	Organization		
	UKA	UKK	UMG
Missing of mandatory data items	4	2	3
Missing of mandatory data values	8	1,748	518
Incomplete inpatient records	1	819	237
Missing of OCs	4	2	11
Implausible ICD-10-GM/OC links	10	3	22
Outlier issues	3	2	8
Ambiguous RD cases	11	3	25
Duplicated RD cases	1	1	3

Abbreviations: DQ, data quality; ICD-10-GM, International Classification of Diseases and Related Health Problems, 10th revision, German Modification; OC, Orphacodes; RD, rare disease; UKA, University Hospital RWTH Aachen; UKK, University Hospital Cologne; UMG, University Medical Center Göttingen.

#### Experiment Settings for Distributed Data Quality Assessments

In order to test and validate our implementation, we first invited three hospitals from the CORD-MI consortium to join the distributed DQ assessments. The participating institutions are University Hospital RWTH Aachen (UKA), University Hospital Cologne (UKK), and University Medical Center Göttingen (UMG). Each hospital set up a single PHT station as well as a FHIR server for synthetic RD patients. We used the station software and On-Boarding workflow 44 to set up the required IT infrastructure for PHT. We also implemented the algorithms as a Docker image to run the DQ analysis in a distributed way as explained above. Next, we configured the train route for participating in the distributed DQ assessments as shown in Fig. 2 .

In addition, we prepared and transformed the synthetic RD data into FHIR bundles including four types of FHIR resources called organization, patient, encounter, and condition. We first developed FHIR tools for extracting the original data from the MII FHIR server 47 and creating FHIR bundles of around 1,000 patients for each participating hospital. The resulting FHIR collection bundles were stored in JSON files that represent three data sets of different organizations namely UKA (Cynthia), UKK (Bapu), and UMG (Airolo). Each data set includes common data items that capture information about the basic modules of the MII-CDS as specified in the FHIR implementation guide of CORD-MI. 39 In this context, we would like to emphasize that when applying our methodology to real-world data, ETL processes have to be implemented that extract the clinical data sets from different data sources of local HIS and transfer them into FHIR resources.

Next, we randomly added DQ issues in these data sets such as duplication, outliers, and implausible RD codification. Table 6 displays the distribution of DQ issues over the three hospitals. For example, the UMG synthetic data set contains 997 cases in which three duplicated RD cases and eight outliers (e.g., age above 115) were randomly introduced. Furthermore, we transformed the FHIR bundles into actionable transactions and distributed them over all participating hospitals. We also developed a python script for enabling an easy upload of created transactions to the FHIR server of each location. The modified data sets are then stored on the local FHIR servers, and in the following, we denote the different FHIR servers by their data set, for example, the Ariolo FHIR server at UMG. The tools and data sets used for data curation are available on GitHub. 42

**Fig. 3:**
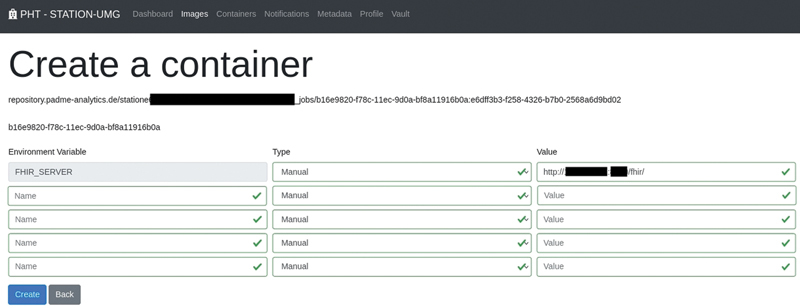
Setting the address of the target FHIR server using the station software of PHT. FHIR, Fast Healthcare Interoperability Resources; PHT, Personal Health Train.

Finally, we started the distributed DQ assessments using PHT. The developed train travels from one station to another to execute including algorithms for evaluating the quality of data stored in the local FHIR servers. If the execution at one station was successful, the train could visit the next station. The stations are used for executing the DQ assessment in a distributed way. The installed FHIR servers are linked to the PHT stations as shown in Fig. 3 . In Section “Distributed Data Quality Assessments”, we present the results of distributed DQ assessments carried out using these experimental settings.

#### Experiment Settings for Local Data Quality Assessment

**Fig. 4:**
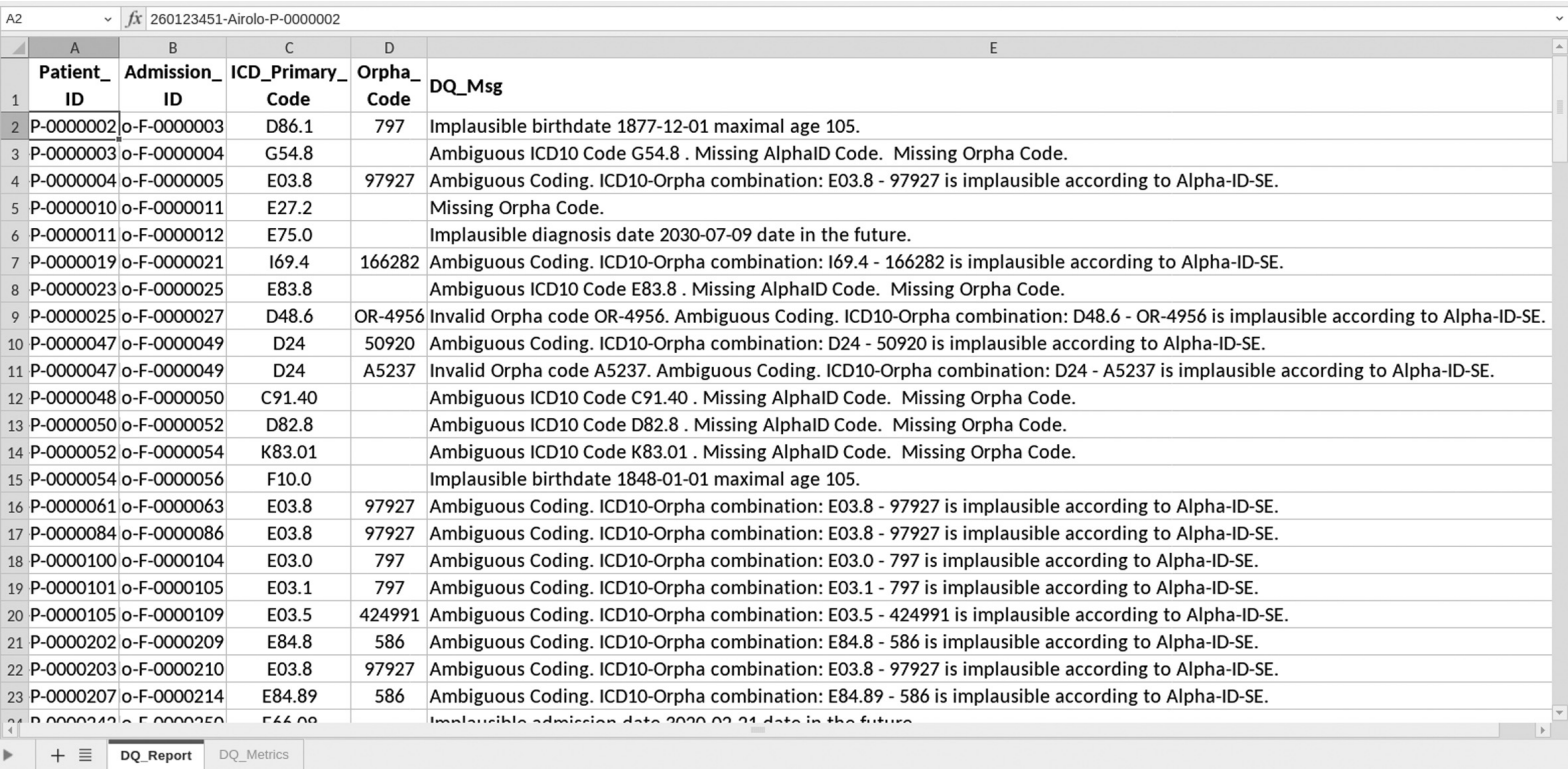
Report on DQ violations detected by DQ assessment of synthetic data stored in the Airolo FHIR server. Abbrevations: DQ, data quality; FHIR, Fast Healthcare Interoperability Resources.

We used the Airolo data set in CSV and Excel formats as well as the Airolo FHIR server installed at UMG for testing the local DQ assessment and validating the obtained DQ results. We would like to note that the number of available inpatient cases, for example, in the Airolo FHIR server in 2020 represents all inpatient cases captured at UMG this year. In contrast to the distributed DQ assessment, local DQ assessment generates the report comprising two Excel spreadsheets. The first sheet also called “report on DQ metrics” illustrates the same DQ metrics as the distributed DQ assessment, while the second sheet also called “report on DQ violations” reports the detected DQ issues, which is an additional function provided only for local execution. To enable users to find the DQ violations and causes of these violations, the second report provides sensitive information such as patient identifier (ID) or case ID (see Fig. 4 ). This reporting function however is only available for local execution in order to meet the data privacy requirements. To validate our implementation, we investigate whether there is a discrepancy between the first and second spreadsheets. In addition, we analyze the concordance between the resulting DQ metrics obtained using distributed DQ assessments and those obtained using local executions on different data formats. In Section “Local Data Quality Assessment”, we present the results of DQ assessments carried out locally using these experimental settings and validation methods.

## Results

Our methodology provides both conceptual and software frameworks that enable a harmonized DQ assessment at single-site and cross-site fashion. Four independent quality dimensions have been proposed in the conceptual framework, namely completeness, plausibility, uniqueness, and concordance. Based on these top dimensions, 9 DQ concepts, 10 DQ indicators, and 25 DQ parameters were defined as shown in Tables 4 and 5 . The implemented software framework provides interoperable tools for calculating required quality metrics and generating local as well as cross-institutional reports on DQ. In this section, we first present the resulting DQ reports as a proof of concept before we demonstrate that our methodology is capable of detecting DQ issues such as outliers or implausibility of coded diagnoses. The results of distributed DQ assessments and local DQ assessments are presented below.

### Distributed Data Quality Assessments

The train first visited UKA, UKK, and finally stopped in UMG with no errors. As a result, distributed analysis was computed over three university hospitals for assessing the quality of FHIR data stored in different HISs as described in Section “Experiment Settings for Distributed Data Quality Assessments”. The generated reports and used tools can be downloaded from GitHub repository. 37 Table 7 illustrates the most important results of DQ parameters that were displayed in the generated DQ reports. For example, Table 7 shows that the UKA data set includes the highest relative frequency of RD cases as well as Orpha cases, while the UMG data set has the highest relative frequency of tracer cases. In this context, we would like to note that the relative case frequencies presented in Table 7 are normalized to 100,000 inpatient cases. The total number of inpatient cases at UKA was 1,000 in 2020. Therefore, 132 cases of those inpatient cases were RD cases, 11 cases were coded with tracer diagnoses, and 128 cases were coded with OCs, that is, 7 tracer cases were coded using ICD-10-GM/OC links.

**Table 7:** Distribution of DQ assessments over the three German hospitals (UKA, UKK, and UMG), DQ parameters for the report year 2020 on synthetic RD data

DQ parameter	Organization		
	UKA	UKK	UMG
ipatCase	1,000	1,000	997
ipat	949	946	950
rdCase_rel	13,200	1,700	10,030
orphaCase_rel	12,800	1,600	9,027
tracerCase_rel	1,100	400	1,906
im_misg	4	2	3
vm_misg	8	1,748	518
ipat_inc	1	819	237
oc_misg	4	2	11
v_ip	3	2	8
link_ip	10	3	22
rdCase_amb	11	3	25
rdCase_dup	1	1	3

Abbreviations: DQ, data quality; RD, rare disease; UKA, University Hospital RWTH Aachen; UKK, University Hospital Cologne; UMG, University Medical Center Göttingen.

The generated reports also include DQ assessments for the top dimensions introduced in the conceptual framework as described in the “Methods” Section. Table 8 gives the resulting DQ indicators obtained for evaluating the completeness, plausibility, uniqueness, and concordance dimensions in each location. We would like to mention that individual DQ indicators are never absolute but should always be seen in the related context and dimensions. Table 8 shows, for example, that the UKA data set is in full agreement with reference values from the literature and yields the best results on the most indicators such as Orphacoding Completeness Rate and Orphacoding Plausibility Rate. However, the Data Item Completeness Rate achieved the worst results with this data set. Moreover, Table 8 also shows the independence of used DQ metrics, for example, although the indicator for data item completeness achieved the best result with UKK data, we got the worst result when assessing the subject or case completeness using the same data set. We even found that while the completeness indicator increases over 80% when assessing the completeness of data values available in the UKK data set, it is reduced by around 13% when evaluating the completeness of inpatient records.

**Table 8:** Distributed DQ assessments over the three German hospitals (UKA, UKK, UMG) using synthetic RD data of year 2020, report on data quality indicators (DQIs) for the top dimensions: completeness, plausibility, uniqueness, and concordance

Top dimension	DQI	Organization		
		UKA	UKK	UMG
Completeness (co)	dqi_co_icr	71.43%	85.71%	78.57%
	dqi_co_vcr	99.92%	85.45%	96.22%
	dqi_co_ccr	66.62%	58.40%	62.14%
	dqi_co_scr	99.89%	13.42%	75.05%
	dqi_co_ocr	63.64%	60%	45%
Plausibility (pl)	dqi_pl_opr	92.19%	81.25%	76.34%
	dqi_pl_rpr	99.92%	99.94%	99.83%
Uniqueness (un)	dqi_un_cur	91.67%	82.35%	75%
	dqi_un_cdr	99.25%	94.44%	97.09%
Concordance (cc)	dqi_cc_rvl	1	0	0

Abbreviations: RD, rare disease; UKA, University Hospital RWTH Aachen; UKK, University Hospital Cologne; UMG, University Medical Center Göttingen.

We tested all types of DQ issues required for CORD-MI as shown in Table 6 and measured the precision and recall values. The developed methods were able to detect all types of randomly introduced DQ issues that were distributed as in Table 6 . Our methodology yields therefore high precision and recall values up to 100%. We also repeated the execution of our algorithm several times with different distributions of random DQ issues in UMG, UKK, and UKA and got the same validation results. Hence, the resulting DQ parameters and indicators validated the correctness and accuracy of performed DQ assessments.

### Local Data Quality Assessment

We applied the developed methods on the Airolo FHIR server located at UMG in order to validate the local and distributed DQ assessments as explained under “Experiment Settings for Local Data Quality Assessment”. As result, two DQ reports were generated automatically (see GitHub repository 37 ). The first report illustrates the calculated DQ metrics, while the second one reports on DQ violations. Fig. 4 shows the DQ issues detected in the second report. The comparison with the report on DQ metrics did not show any discrepancy between the displayed DQ issues and calculated DQ metrics. The generated report on DQ violations is used to establish iterative feedback to potential users and to improve the quality of RD data. Potential users are for example medical documentation assistants or data scientists. We would like to mention that, if applied to real-world data, the spreadsheet for DQ violations cannot be shared as it contains sensitive information that may be traced back to individual patients.

Besides the spreadsheet for DQ violations, the generated reports also provide adequate information about the quality metrics calculated for the top dimensions: completeness, plausibility, uniqueness, and concordance. The obtained results are in full agreement with distributed DQ assessments shown in Tables 7 and 8 (UMG). We also repeated the execution of local DQ assessments several times with other formats such as CSV or Excel and got the same validation results. Our methodology yields therefore high precision and recall values up to 100% in both experiments for local and distributed DQ assessments. Further, we demonstrated that distributed DQ analysis using PHT can achieve assessment results as good as local execution.

## Discussion

The developed methods were successfully tested locally and distributed across multiple hospitals. The proposed conceptual and software frameworks provide an interoperable solution for privacy-preserving assessments of DQ. We have presented a proof of concept study that demonstrates the capability of our software framework to perform DQ analysis on decentralized data sources distributed among the Germany-wide CORD-MI consortium. Our study has shown that the proposed methodology is capable of detecting potential DQ issues and that it can be used for local as well as cross-institutional reporting on DQ. The DQ reports can help users to find the causes of detected DQ violations as shown in Fig. 4 . The developed DQ metrics are useful and cover independent aspects of DQ as presented in the “Results” Section. We used precision and recall to test and validate the implementation of our distributed DQ assessments. As a result, our methods yield high precision and recall values up to 100% in local and distributed DQ assessments. We also repeated the execution of our algorithm several times with different distributions of random DQ issues and got the same validation results. Our implementation is therefore generic and does not depend on the configuration of build-in DQ issues. However, we would like to emphasize that the quality issues have not been introduced by an independent party and may therefore be regarded as a validation of the implementation rather than a performance measure. Essentially, the developed DQ checks and metrics meet the requirements that were elicited during the system design process together with the domain experts as mentioned in Section “Data Quality Challenges and Requirements”. Hence, our methodology provides a useful solution for evaluating the quality of distributed RD data that considers user and domain-specific requirements. Moreover, the proposed software framework enables a harmonized and interoperable assessment of DQ that does not depend on local configuration and HIS architectures. Our methodology can therefore be used for DQ benchmarking to improve the quality of RD documentation. DQ indicators should however not be misused for benchmarking purposes. We have shown that individual DQ indicators are never absolute and should be seen in the overall context and full picture of different DQ dimensions shown in the ontology presented in Fig. 1 .

DQA tool, 27 DQ Dashboard, 28 and Achilles Heel 48 also provide tools for DQ assessment but strongly follow the Observational Medical Outcomes Partnership (OMOP) Common Data Model (CDM) specifications and are therefore not applicable for data stored in the FHIR standard or for distributed DQ assessments on RD data. The approach by Tute et al 10 also proposed a centralized DQ assessment that relies on the open Electronic Health Record (OpenEHR) data model, and as a consequence, is only applicable to openEHR-based data sources. Another limitation of this approach is that it does not investigate the uniqueness and concordance dimensions. It focuses on the completeness and plausibility of data values.

In accordance with other well-known theoretical approaches such as those proposed by Kahn et al 11 and Schmidt et al, 12 completeness and plausibility represent the main dimensions of DQ. However, our approach covers other important DQ dimensions such as concordance and semantic uniqueness. The latter is especially necessary for enabling the secondary use of clinical data. Moreover, our conceptual framework introduces other facets of completeness and plausibility that are required for assessing the DQ in CORD-MI. Hence, the developed methods differentiate data item completeness from data value completeness as described in the “Methods” Section, allowing specifically for assessing completeness according to an external reference information model. In this context, we also distinguish between subject completeness, module completeness, and vector completeness as shown in the ontology presented in Fig. 1 . Our DQ concept provides therefore more specific indicators for assessing the value completeness dimension such as Subject Completeness Rate, Case Completeness Rate, and Orphacoding Completeness Rate. Furthermore, the extension of plausibility by the semantic plausibility dimension allows us to define more specific metrics such as the Plausibility Rate of Orphacoding.

In addition to the reporting of DQ indicators that are provided by a number of practical approaches proposed in the literature, we implemented a detailed report on detected DQ issues to establish an interactive DQ improvement process, requested by the domain experts. Both requirements are necessary and have been therefore considered in our methodology. Another advantage of our software framework is that it also follows the modular design that allows users to select desired parameters and indicators as well as to generate specific reports on DQ.

The DQ indicators proposed in this work are to a large extent specific for assessing data recorded across CORD-MI hospitals. However, the first 11 DQ parameters (P1,..,P11) presented in Table 4 are generic and can therefore be reused for different data sets that follow the MII-CDS specifications. In addition, a number of DQ indicators such as the completeness and plausibility indicators can also be applied to other MII use cases. Moreover, our methodology provides a generic ontology that covers different categories of DQ dimensions as shown in Fig. 1 . Hence, this ontology can be reused for the classification of existing DQ metrics or for defining new indicators specific to other domains.

Besides PHT, there have been multiple approaches proposed and applied for analyzing sensitive data without explicitly sharing it, such as DataSHIELD 49 and Secure Multiparty Computation (SMPC). 50 In DataSHIELD, Opal servers are running on each data holding organization and researchers use a fixed R-library to execute the analysis requests on the Opal servers. SMPC is a cryptographic protocol that enables joint computation of a function across multiple parties where no individual party can see the data from other parties. Significant communication overhead is the main challenge of SMPC being applied to more complex functions, for example, in machine learning. Compared to other methods, the advantages of PHT are flexibility with data sources, for example, FHIR or DICOM, and programming language agnostic. Moreover, PHT is OS-independent and scalable due to the characteristics of containerization technologies.

## Limitations

Our software framework currently does not provide DQ metrics for specific domains such as cardiology or omics. More collaborations with experts are therefore needed to expand this framework and define new indicators that fit well within other domain requirements. The developed software framework uses Alpha-ID-SE as a reference for assessing the quality of Orphacoding. This terminology does not cover all OCs provided by Orphanet and primarily focuses on the disorder level. However, the current version of Alpha-ID-SE includes the most relevant RD and will be yearly updated with new codes and labels of RD diagnoses on the disease entity level. The 2022 release will be quite close to full coverage of Orphanet. We acknowledge that the quality assessment of Orphacoding based on the Alpha-ID-SE is a political decision. This is also due to the legally required Orphacoding in German hospitals which needs to be implemented starting April 1, 2023. 26

Our software framework uses FHIR as a gold standard and does not support other standards such as OMOP-CDM and OpenEHR. However, several tools have been provided in the literature for automated mapping of OpenEHR and OMOP data sets to FHIR. 51 52 The application of our methods is therefore not limited to local data models or HIS architectures. Nevertheless, heterogeneous data sources have to be transformed into FHIR resources using ETL and FHIR mapping processes. Such processes are usually time-consuming and expensive. To enable an easy import of data directly exported from HIS data sources, our implementation also supports user-friendly spreadsheet formats such as Excel and CSV.

We used fhircrackr package 45 to develop a FHIR interface that enables interoperable DQ assessments as described in the “Methods” Section. However, this package does not provide functions to determine whether empty data vectors are due to missing metadata in a given FHIR data set or due to missing data values. The latter case means that in the whole data set not a single value to the related data items was collected. This is possible but not very likely. Hence, empty data vectors are considered as missing data items in the current implementation, and as consequence, do not influence the indicator for value completeness (dqi_co_vcr) presented above.

The proposed DQ concept is implemented in R and available on GitHub. 37 41 The functionality of R code usually depends on the version of used packages. To avoid local dependency issues, we provide a docker environment that can be started by one command to run the DQ assessment and generate specific reports. When applying our implementation to real-world data in future analyses, the second report about detected DQ issues can currently not be published or shared with other DICs, because it contains patient-related information. In future work, we will use anonymization techniques to remove sensitive information and include this report in distributed DQ assessments as well. We will also extend our software framework with visualization methods to improve the usability of proposed tools for DQ assessment.

## Conclusion

The developed conceptual and software frameworks provide valuable DQ metrics and an interoperable solution for evaluating the DQ of RD documentation in distributed data sources of CORD-MI. As proof of concept, we applied this methodology to synthetic data stored in different HISs. In effect, the developed DQ checks and metrics meet the requirements that were elicited during the system design process. As a result, our study has demonstrated that our methodology can detect randomly introduced DQ issues such as missings, outliers, ambiguity, and implausibility of coded diagnoses and that it can be applied for cross-site reporting on DQ. We successfully validated the implementation of our distributed DQ assessments using PHT. The test results have indicated the correctness and accuracy of calculated indicators and parameters. The implemented DQ metrics are flexible and cover independent aspects of DQ. The developed frameworks provide useful methods for a harmonized and privacy-preserving assessment of DQ. Our methodology therefore meets the specified requirements and can be used for DQ benchmarking on real-world data to improve the quality of RD documentation as well as to support clinical research on distributed data sources.
